# Identification and Analysis of Mitogen-Activated Protein Kinase (MAPK) Cascades in *Fragaria vesca*

**DOI:** 10.3390/ijms18081766

**Published:** 2017-08-13

**Authors:** Heying Zhou, Suyue Ren, Yuanfang Han, Qing Zhang, Ling Qin, Yu Xing

**Affiliations:** Beijing Key Laboratory for Agricultural Application and New Technique, College of Plant Science and Technology, Beijing Collaborative Innovation Center for Eco-Environmental Improvement with Forestry and Fruit Trees, Beijing University of Agriculture, Beijing 102206, China; zhydyx2012@126.com (H.Z.); fenhui315@126.com (S.R.); hanyuanfang186@163.com (Y.H.); zhangqing@bua.edu.cn (Q.Z.); qinling@bua.edu.cn (L.Q.)

**Keywords:** *Fragaria vesca*, MAP kinase cascades, phylogenetic relationships, abiotic stresses, gene expression

## Abstract

Mitogen-activated protein kinase (MAPK) cascades are highly conserved signaling modules in eukaryotes, including yeasts, plants and animals. MAPK cascades are responsible for protein phosphorylation during signal transduction events, and typically consist of three protein kinases: MAPK, MAPK kinase, and MAPK kinase kinase. In this current study, we identified a total of 12 *FvMAPK*, 7 *FvMAPKK*, 73 *FvMAPKKK*, and one *FvMAPKKKK* genes in the recently published *Fragaria vesca* genome sequence. This work reported the classification, annotation and phylogenetic evaluation of these genes and an assessment of conserved motifs and the expression profiling of members of the gene family were also analyzed here. The expression profiles of the *MAPK* and *MAPKK* genes in different organs and fruit developmental stages were further investigated using quantitative real-time reverse transcription PCR (qRT-PCR). Finally, the *MAPK* and *MAPKK* expression patterns in response to hormone and abiotic stresses (salt, drought, and high and low temperature) were investigated in fruit and leaves of *F. vesca*. The results provide a platform for further characterization of the physiological and biochemical functions of MAPK cascades in strawberry.

## 1. Introduction

Plants are influenced by different biotic or abiotic stresses, thus in response have developed a few signaling networks to protect them at the molecular or cellular levels [1]. A series of inter-lined regulatory signaling networks is composed of some stress-activated molecular pathways [2]. The protein-kinase-based cascades associated with responses to extracellular stimuli are the mitogen-activated protein kinase (MAPK) cascades, which are conserved components of signal transduction pathways in eukaryotes ranging from yeast to plants and animals [3,4,5,6].

MAPK cascades involve protein phosphorylation events that contribute to signaling [3], and acknowledged eukaryotic MAPKs can be separated into three main subfamilies according to their structural characteristics, which is often related to their functions in various signal transduction pathways. The MAPK cascades classically are composed three protein kinases, MAPK (MAPK/MPK), MAPK kinase (MAPKK/MKK), and MAPK kinase kinase (MAPKKK/MAP3K), but sometimes contain a MAP3K kinase that phosphorylates the corresponding downstream substrates [7,8]. MAPK can catalyze the phosphorylation of a substrate protein by chemically adding phosphate groups from adenosine triphosphate (ATP) [9]. MAP3Ks are substantially serine or threonine kinases that phosphorylate MAP2Ks at a conserved S/T-X3−5-S/T motif [10,11,12]. MAPKs have TDY and TEY phosphorylation motifs in their activation loops, which can be phosphorylated by MAPKKs [1,9]. Plant MAPKs can be separated into four groups (A, B, C and D), in which members of Groups A, B and C possess the TEY motif at its phosphorylation site, and members of Group D possesses the TDY motif [1].

In plants, MAPKs have been shown to phosphorylate different substrates, such as cytoskeleton binging proteins, transcription factors, and other kinases [3,13]. Many genes that encode MAPK cascades have been characterized from *Arabidopsis thaliana*, tobacco (*Nicotiana tabacum*), barley (*Hordeum vulgare*), maize (*Zea mays*), and rice (*Oryza sativa*) [10,12,14,15]. In *A. thaliana*, a complete MAPK cascade (MEKK1-MKK4/5-MAPK3/6) triggered by bacterial flagellin-derived peptide flg22 was characterized, which up-regulated the expression of the transcription factors of WRKY22/29 and then increased resistance to both fungal and bacterial pathogens [16]. CTR1 (the RAF-like MAP3K) plays an important role in leaf senescence and camalexin biosynthesis [17,18]. Many reports showed that MAPK cascades involved in different stress responses, such as drought, salt, cold or ABA treatment in different crops [19,20,21,22,23,24,25,26,27,28,29]. MKKs containing 11 catalytic subdomains are identified by a kinase domain and a K/R-K/R-K/R-X1-6-L-X-L/V/S MAPK-docking domain, and are functioned by phosphorylated their conserved serine and threonine residues (S/T-X3-5-S/T motif) [5]. MKKs can be classified into four groups according to the description in *A. thaliana* and rice, and several MKKs have been characterized in a variety of plant species [30]. Those MKKs are involved in several abiotic stresses. In *A. thaliana*, MKK1 mediated ABA induced *CAT1* expression during drought stress; the *FSD2/3* expressions were activated by MEKK1 via MKK5-MPK6-coupled signaling in salt stress; and MKK5 involved into high light induced CSD expressions [20,21,22,23]. SIPK and WIPK activated by NtMEK2 influenced cell death [31], and SIMKK played a key role in both salt- and elicitor-induced signals in alfalfa (*Medicago sativa*) [32,33].

As the largest class of MAPK cascades, the MAP3Ks can be divided into three subfamilies (MEKK, Raf and ZIK). A conserved G(T/S)Px(W/Y/F)MAPEV kinase domain can be found in the members of the MEKK subfamily, and in the ZIK subfamily, most of the proteins have the GTPEFMAPE(L/V)Y domain, while Raf subfamily members have the GTxx(W/Y)MAPE domain [8]. The members of the RAF subfamily have a kinase domain in C-terminal and a long regulatory domain in their N-terminal, compared with the members in the ZIK subfamily, which have the kinase domain in the N-terminal [34]. In *A. thaliana*, MEKK1 has been shown to regulate defense responses against bacteria and fungi [35], and a Raf-like MAP3K, AtEDR1, is involved in SA-inducible defense responses [36]. The ZIK subfamily, which contains 10 and 9 members in *A. thaliana* and rice, respectively, regulates flowering time and circadian rhythms [37].

The identification and characterization of different members of the MAPK cascades have been reported by functional genomic studies in various plant species, including *A. thaliana*, maize, rice, alfalfa, tobacco and grapevine (*Vitis vinifera*). They comprise complex gene families, which have been best studied in model plants, such as *A. thaliana* and rice [1,3,5,38,39,40,41]. There are at least 80 *MAPKKKs*, 10 *MAPKKs* and 20 *MAPKs* in the *A. thaliana* genome, whereas the rice genome contains at least 75 *MAPKKKs*, 8 *MAPKKs* and 17 *MAPKs*. Few MAPK cascades have been identified in fruit crops, although 14 *MAPKs*, 5 *MAPKKs*, 62 *MAPKKKs* and 7 *MAPKKKKs* were identified in the grapevine genome, and less is known in their regulatory mechanism and function. This is especially true for many fruit crops of horticultural value.

It is unknown in their functions of most MAPK in plants, but MAPK cascades have been identified in horticultural crops, such as grapevine and cucumber (*Cucumis sativus*) [34,42]. In the current study, we identified a total of 12 *FvMAPKs*, 7 *FvMAPKKs*, 73 *FvMAPKKKs* and 1 *FvMAPKKKK* from the recently published strawberry (*F. vesca* “Hawaii 4”) genome [43]. We report the classification, annotation and a phylogenetic evaluation of these genes, and an assessment of conserved motifs and the results of expression profiling of members of the gene family were also analyzed here. The expression patterns of *MAPK* cascades genes in different organs and different fruit developmental stages were further analyzed using quantitative real-time reverse transcription PCR (qRT-PCR), and the gene expression patterns in response to hormone and abiotic stress in leaves and fruit were also assessed. The results may provide information for further functional studies of this kind of kinases in strawberry.

## 2. Results

### 2.1. Genome-Wide Identification of MAPK Cascade Genes in F. vesca

A total of 12 MAPK, 7 MAPKK, 73 MAPKKK and 1 MAP4K open reading frames (ORFs) encoding putative MAPK cascade proteins were identified in the *F. vesca* genomic [43]. The number of *FvMAPK* genes (12) was less than in *A. thaliana* (20) and rice (17). Among them, the lengths of the deduced proteins ranged from 365 amino acids (FvMPK9) to 691 residues (FvMPK3), and the pI values ranged from 5.05 (FvMPK9) to 9.26 (FvMPK2). The number of genes in the MAPKK subfamily (7) was even lower than in A. thaliana, which has 10 members. In this subfamily, the full lengths of the protein sequences ranged from 244 to 518 amino acids; however, the pI values were similar to the FvMAPK proteins and ranged from 5.34 (FvMAPKK5) to 9.28 (FvMAPKK2). Among the 73 MAPKKKs, the pI values ranged from 4.50 to 10.09 (Table 1).

**Figure 1:**
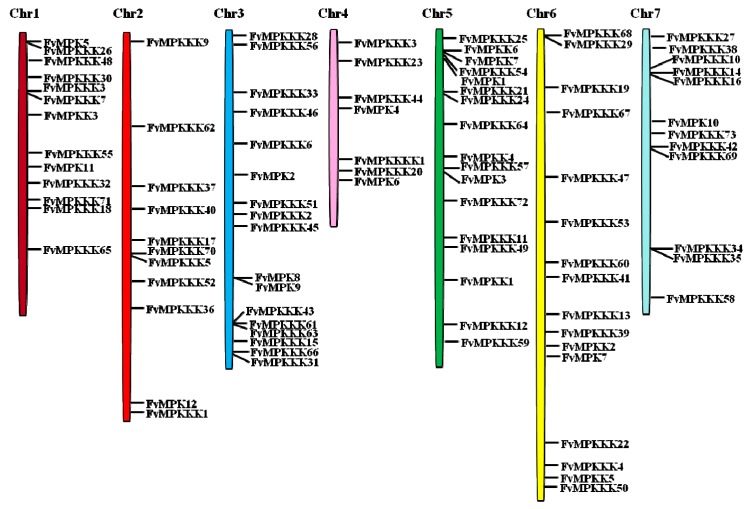
Chromosomal locations of strawberry *MAPK*, *MAPKK*, *MAPKKK* and *MAP4K* genes. Chr1–Chr7 represent the seven chromosomes. Black lines on bars indicate the locations of each gene.

To investigate the genetic divergence within the *MAPK* cascade genes as well as the gene duplication patterns, the physical locations of the genes on the strawberry chromosomes were investigated (Figure 1). Ninety-three genes were mapped on all seven chromosomes, with distributions from ranging from 11 to 17. Chromosomes 3 and 5 contained 17 *MAPK* cascade genes, and chromosomes 6, 1, 7, 2 and 4 contained 16, 13, 12, 11 and 7 genes, respectively.

**Figure 2:**
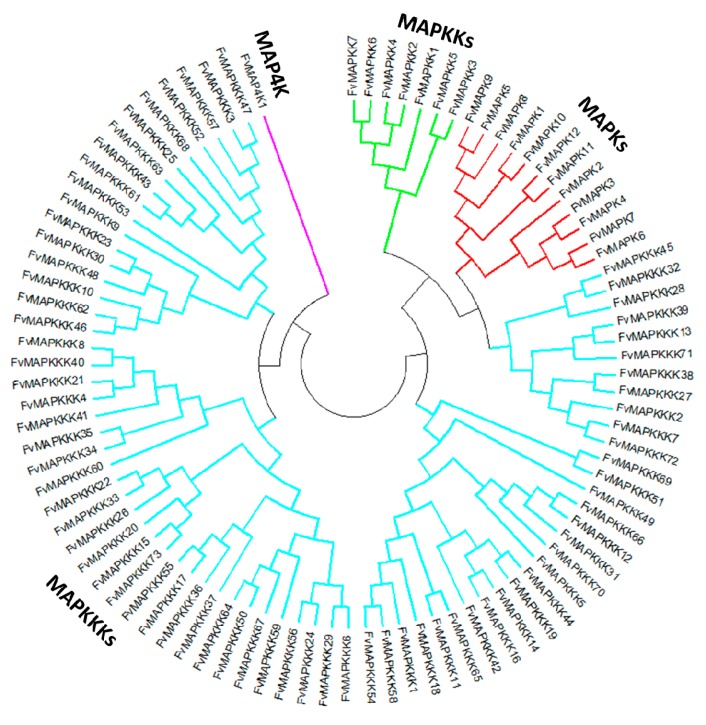
Phylogenetic relationships between strawberry MAPK cascade proteins. The abbreviations of MAPK cascade proteins are as follows: MAPK, Mitogen-activated Protein Kinase, red lines; MAPKK, MAPK Kinase, green lines; MAPKKK, MAPKK Kinase, blue lines; MAP4K, MAPKKK Kinase, purple lines.

**Figure 3:**
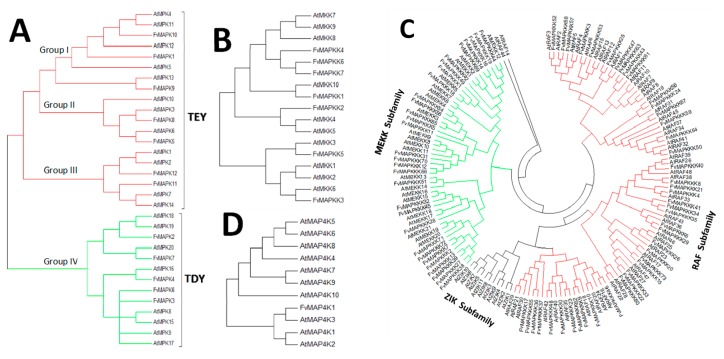
Phylogenetic relationships between MAPK cascade proteins from Arabidopsis thaliana and strawberry: (**A**) phylogenetic relationship between *A. thaliana* and FvMAPK proteins, red lines show TEY group; (**B**) phylogenetic relationship between *A. thaliana* and FvMAPKK proteins, green lines show TDY group; and (**C**) phylogenetic relationship between *A. thaliana* and FvMAPKKK proteins. MAPKKK forms the largest group of MAPK cascade proteins with 73 members classified into two subfamilies, MEKK (green lines) and Raf (red lines), which contain 30 and 43 genes in the *Fragaria vesca* and *A. thaliana* genomes. (**D**) Phylogenetic relationship between *A. thaliana* and strawberry MAPKKKK proteins.

A phylogenetic tree was generated to provide insights into the evolutionary divergence of the MAPK cascade from a common ancestor, and to infer evolutionary relationships among various genes or species. All predicted MAPK cascade protein sequences and the corresponding A. thaliana sequences were aligned, and a rooted phylogenetic tree was constructed by aligning full length amino acid sequences (Figure 2 and Figure 3). The FvMAPK sequences were further divided into four subfamilies based on the conserved threonine and tyrosine residues in the TEY and TDY motifs in their phosphorylation activation loop (Figure 3A). Only one MAP4K was identified, and this was predicted to contain the conserved TFVGTPxWMAPEV motif.

The division of the FvMAPK sequences into four distinct groups was consistent with previous reports [14,37]. FvMAPK1, FvMAPK9 and FvMAPK10 clustered within Group I. MAPKs are involved in both abiotic stress responses and cell division in A. thaliana [23,24]. FvMAPK5 and FvMAPK8 belonged to Group II, together with AtMPK3 and AtMPK6, and Group III includes FvMAPK11 and FvMAPK12 and members from this group in other plant species are known to be regulated by both biotic and abiotic stresses [25]. The largest group was Group IV, which included five members (FvMAPK2, FvMAPK3, FvMAPK4, FvMAPK6 and FvMAPK7) with the TDY motif in their T-loop and the absence of the C-terminal CD domain, which is consistently present in members of the other MAPK groups.

Seven members of the MAPKK subfamily, similar to the AtMAPKKs, could also be divided into four groups: FvMAPKK1, FvMAPKK3 and FvMAPKK5 were most similar to AtMPKK10, AtMPKK6 and AtMPKK3, respectively, while FvMPKK2 is homologous to AtMPKK4/AtMPKK5. FvMPKK4, -6 and -7 and AtMPKK7, -8, and -9 clustered into the same group (Figure 3B).

MAPKKKs are activated by either phosphorylation by other MAPKKK kinases or by G proteins and G protein-coupled receptors. With 73 members, the MAPKKK subfamily represented the largest subfamily, of a similar size to that in rice (75 members), larger than that in grape (62 members), but smaller than that in A. thaliana (80 members). Generally, the members of this subfamily can be classified into three groups: the MEKK subfamily, the ZIK subfamily and the Raf subfamily; however, only two groups were identified in the strawberry genome. In total, there were 30 FvMAPKKKs in the MEKK subfamily, and 43 in the Raf subfamily with no ZIK members identified (Figure 3C).

Although several MAP4Ks have been reported in plant genomes based on phylogenetic analyses of their kinase domain, little is known about the roles of MAPKKKKs in plants. Only one member was identified in *F. vesca* (Figure 3D).

### 2.2. Expression Profiles of FvMAPKs and FvMAPKKs in Different Organs and Fruit Developmental Stages

**Figure 4:**
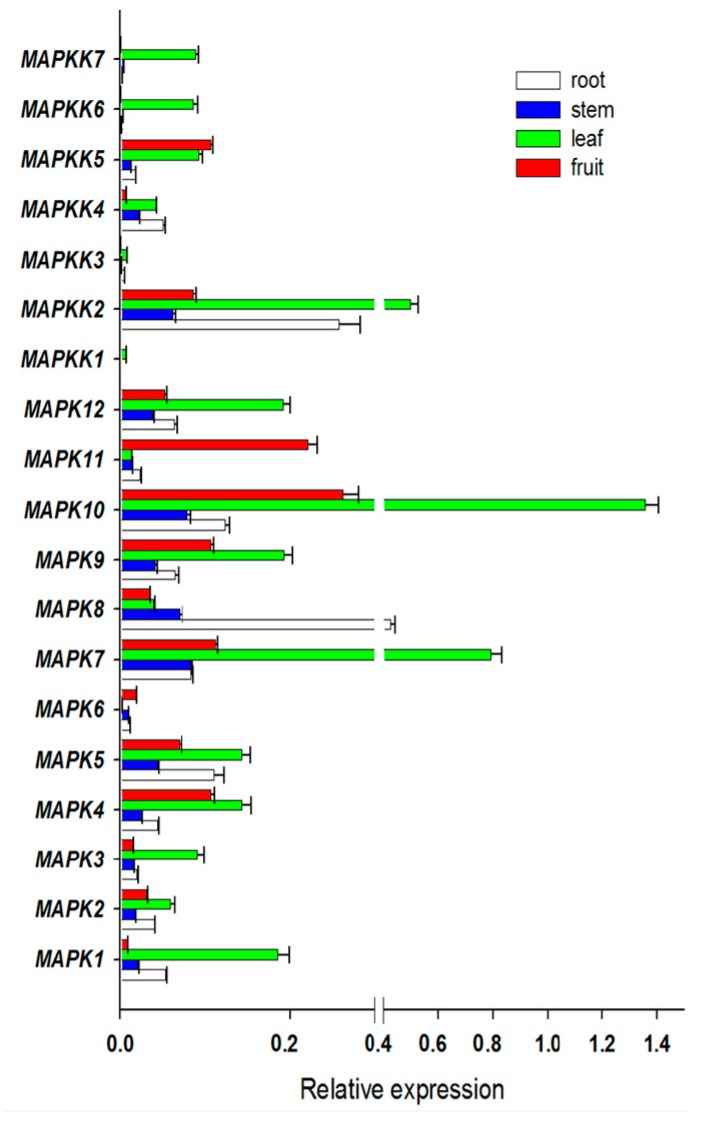
Expression patterns of *FvMPKs* and *FvMPKKs* in different organs. *X*-axis is the relative expression, while *Y*-axis is *MAPK* and *MAPKK* genes; white box, root; blue box, stem; green box, leave; red box, fruit.

Expression profiles provide some useful clues to gene functions. To investigate the putative involvement of *FvMAPK* and *FvMAPKK* genes in strawberry growth and development, the expression patterns of all of these genes were analyzed under normal growth conditions in four different organs (leaves, roots, stems and fruits) at various developmental stages. Of the 19 predicted genes, all were expressed in at least one of the four organs (Figure 4). *FvMPK7* and *FvMPK10* showed high expression in leaves and *FvMPK10* and *FvMPK11* were highly expressed in fruits. *FvMAPKK2*, *FvMAPKK4* and *FvMAPKK5* were expressed in all organs, while *FvMAPKK6* and *FvMAPKK7* were only expressed in leaves, and *FvMAPKK1* and *FvMAPKK3* did not express any of the different organs under normal growth conditions.

**Figure 5:**
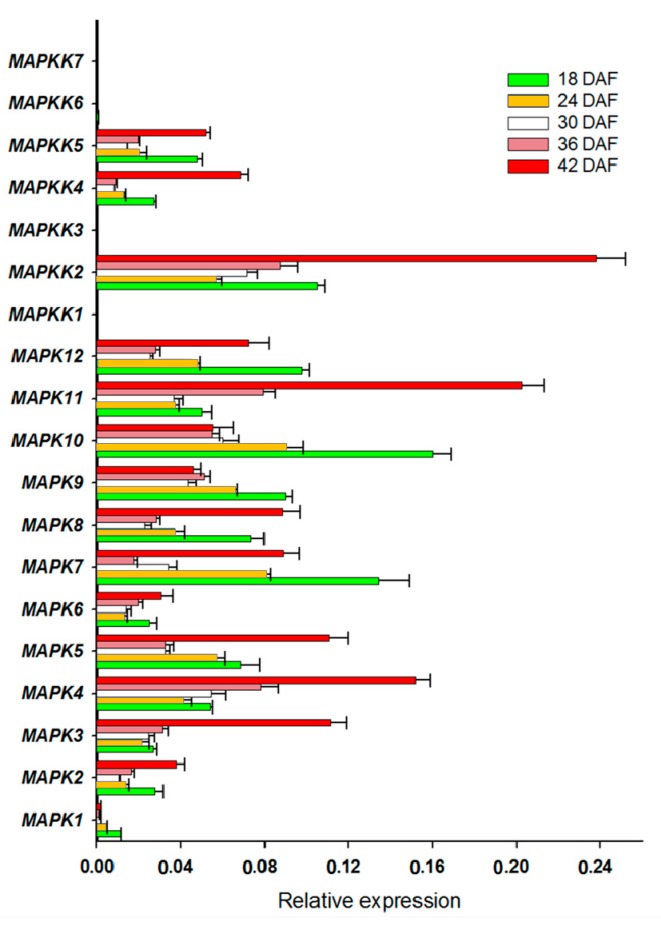
*FvMPK* and *FvMPKK* expression profiles in different fruit developmental stages. *X*-axis is the relative expression, while *Y*-axis is *MAPK* and *MAPKK* genes; 18–42 DAF, five developmental stages of days after flowering.

**Figure 6:**
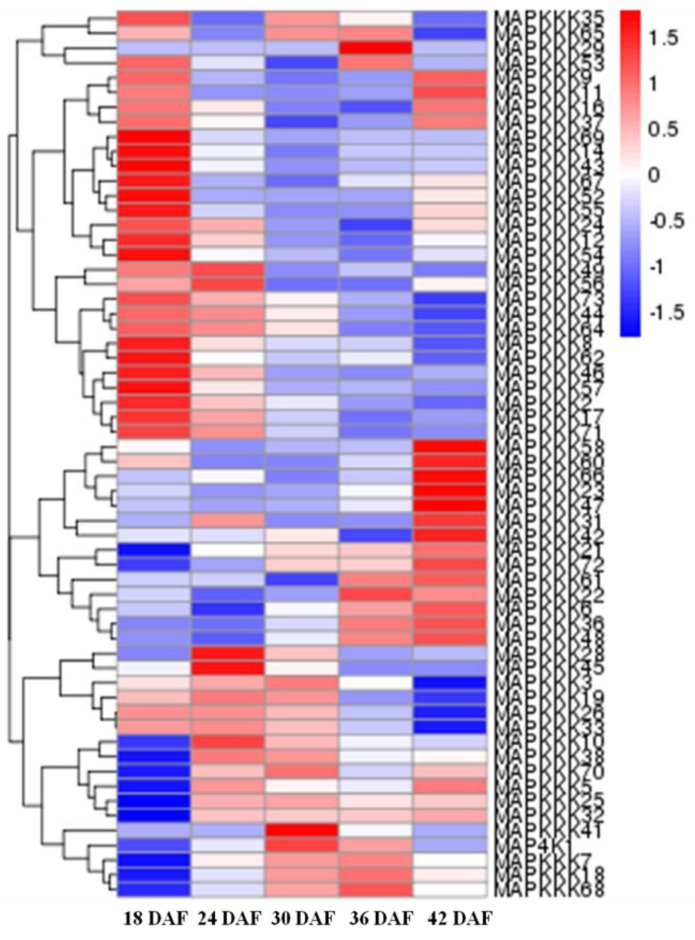
RNA-seq data showing *FvMAPKKK* gene expression in different fruit developmental stages. The gene expression levels were showed by the color box from blue to red indicating from low to high for each gene; 18–42 DAF, five developmental stages of days after flowering.

We also determined the expression of FvMAPK cascade genes in different fruit developmental stages. The transcript levels of *FvMAPK2* and *FvMAPK12* were lower than any other of the genes in all the different fruit developmental stages. For the remaining 10 genes, the expression patterns could be roughly divided into three categories: *FvMAPK3*, *FvMAPK4* and *FvMAPK11* showed the same expression patterns, which increased through development. *FvMAPK1*, *FvMAPK9* and *FvMAPK10* expression, however, declined during fruit development. Finally, there were six genes (*FvMAPK5*, *FvMAPK6*, *FvMAPK7* and *FvMAPK8*) that showed a high expression in the early stages, then a decrease and later an increase in the later stages of fruit development. In the *FvMAPKKs* subfamily, only *FvMAPKK2*, *FvMAPKK4* and *FvMAPKK5* were expressed in fruits at a detectable level, and they had the same expression patterns, which gradually rose through fruit development (Figure 5). Since *F. vesca* is a new model for investigating non-climacteric fruit development and ripening, we focused on determining the expression profiles of the *FvMAPKKK* subfamily to different fruit developmental stages. The expression profiles of the *FvMAPKKK* genes revealed a much higher expression in early stages than in later ripening or senescing stages, indicating that they are most likely related to signal transduction during development in metabolically active tissues (Figure 6). Some FvMAPKKK transcripts showed higher levels in earlier than later stages of fruit development, while others showed the opposite pattern. This information can be important for further investigation of the signal transduction pathways involved in the regulation of fruit development and ripening, in which different MAPKKK subfamily members may be involved.

### 2.3. Gene Expression Profiles of FvMAPKs and FvMAPKKs in Response to Hormones and Abiotic Stresses in F. vesca Leaves

**Figure 7:**
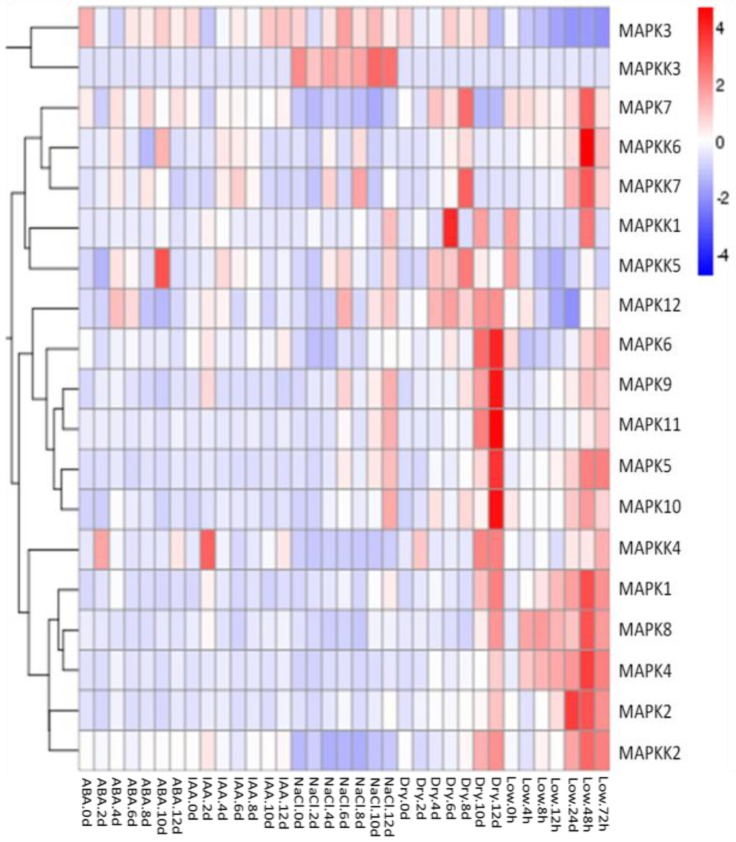
Expression patterns of *FvMPK* and *FvMPKK* genes after different stress and hormone treatments of leaves in *Fragaria vesca*. Leaves were harvested every two days (0, 2, 4, 6, 8, 10 and 12 days) after treatment: Abscisic acid (ABA) (100 μM), indole-3-acetic acid (IAA) (100 μM), NaCl (100 mM) and drought treatments. Leaves from the low temperature treatment were collected at 0, 4, 8, 12, 24, 48 and 72 h after cold stress application.

We investigated whether salt, drought, low temperature and exogenous application of the hormones indole acetic acid (IAA) and abscisic acid (ABA) would induce expression of the *FvMAPK* and *FvMAPKK* genes in seedling leaves using qRT-PCR analysis. After cold treatment, most of the tested genes showed a large expression increase 48 h after treatment. Among them was *MAPK2*, whose expression increased in early stages before decreasing. In general, drought stress resulted in the induction of the greatest number of genes and a significantly improved in the process 12 days. In response to salt stress, the expression of five *FvMAPK* genes (*FvMAPK5*, -*9*, -*10*, -*11* and -*12*) and three *FvMAPKK* genes (*FvMAPKK1*, -*3* and -*5*) increased. Interestingly, the transcript levels of *FvMAPKK3* were specifically activated by salt stress and not by the other treatments. The expression of *FvMAPK3* and -*12* and *FvMAPKK4*, -*5* and -*6* was activated by ABA, while the IAA treatment only resulted in a change in *FvMAPKK4* expression, which was up-regulated two days after treatment (Figure 7).

### 2.4. Expression Patterns of FvMAPK and FvMAPKK Genes in Response to Sucrose, Hormones and Abiotic Stresses in F. vesca Fruits

**Figure 8:**
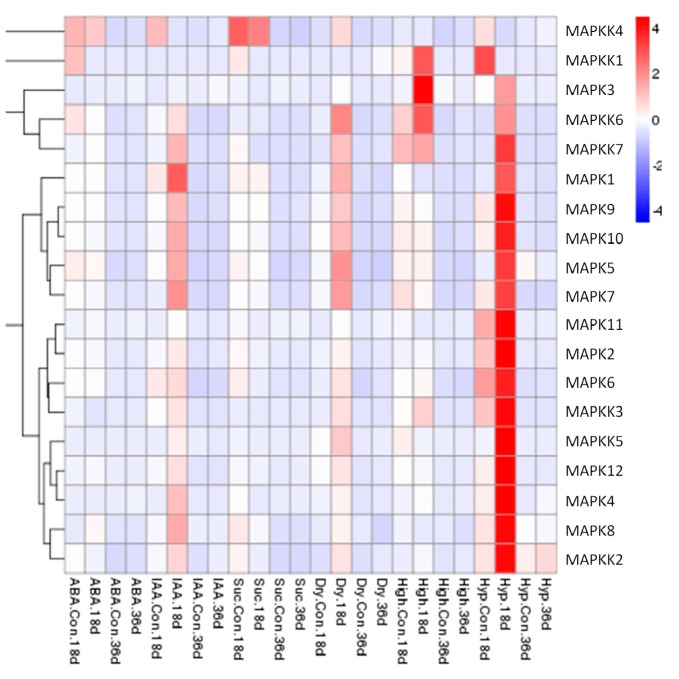
Expression patterns of *FvMPK* and *FvMPKK* genes in fruits after different stress pre-treatments. Red and blue boxes indicate high and low expression levels, respectively, for each gene. Six treatments: Abscisic acid (ABA), 100 μM; indole-3-acetic acid (IAA), 100 μM; Suc, sucrose 50 μM; Dry, drought treatment; High, high temperature (40 °C); Low, low temperature (4 °C); Con., controlled trials of fruits without any pre-treatments; 18d., 18 DAF fruits; 36d., 36 DAF fruits.

The expression patterns of the *FvMAPK* and *FvMAPKK* genes at an early stage (18 DAF: days after flowering) and a late stage (36 DAF) of fruit development in response to exogenous IAA, ABA and sucrose treatment and drought, high temperature or low temperature stresses were also examined by qRT-PCR analyses. Almost all showed a higher expression level in the early than in the later stage. It was interesting that the transcript levels of almost all the genes were up-regulated in response to IAA, drought and low temperature treatment at the early stage of fruit development (18 DAF). During low temperature stress, the expression of 17 of the 19 genes increased significantly at 18 DAF, while the transcript levels of *FvMAPK3*, *FvMAPKK1*, *FvMAPKK3*, *FvMAPKK6*, and *FvMAPKK7* were significantly up-regulated by high temperature treatment, even though the expression of *FvMAPKK1*, *FvMAPKK3*, *FvMAPKK6*, and *FvMAPKK7* could not be detected under normal growth conditions (Figure 8).

## 3. Discussion

Although MAPKs have been studied in other plant species, the FvMAPK cascades have not yet been comprehensively studied. Here, we identified 12 *FvMAPK*, 7 *FvMAPKK*, 73 *FvMAPKKK* and 1 *FvMAPKKKK* genes. There are only 12 *MAPK* and 7 *MAPKK* members in the strawberry genome, while at least 20 *MAPK* genes and 10 *MAPKK* genes have been identified in the *A. thaliana* genome, even though the genome size of *F. vesca* (240 Mb) is approximately twice that of the *A. thaliana* genome (~125 Mb). The numbers are more comparable to those in *V. vinifera* (~400 Mb), where 14 MAPK and 5 MAPKK members have been identified [1,42]. With 73 members, the MAPKKK subfamily is similar in size to that of rice (75 members), larger than that of grapevine (62 members) but smaller than that of *A. thaliana* (80 members). Only a few *MAP4K* genes have been identified in plant genome based on phylogenetic analysis of the kinase domain [5,15]. At least 10 protein kinases can be phylogenetically characterized as MAP4K in the *A. thaliana* and rice genomes, but little is known about their roles. Only one MAP4K member was identified in the *F. vesca* genome, but the ORF showed strong similarity to *ScMAP4K1* from the wild potato species *Solanum chacoense*, which is known to play important roles in ovule, seed, and fruit development [44], and a similar role is therefore possible in strawberry.

The phylogenetic analysis showed that the FvMAPKs can be divided into four groups based on the conserved residues of the TEY/TDY motifs in the activation loop region (T-loop) between the kinase subdomains VII and VIII [9]. Members of the FvMAPK subfamily showed 24–89% identity to each other FvMAPKs, and their full-length sequences ranged from 365 to 691 amino acids. The difference in length is usually due to variation in the length of the domain or a variable number of introns, and may indicate the absence or presence of motifs, which can affect the functional specificity. There are 10 and 7 MAPKK members in *A. thaliana* and *F. vesca*, respectively. The full length FvMAPKK proteins ranged in size from 244 to 518 amino acids, and shared 25–42% similarity with each other. The members of the FvMAPKKK subfamily were distributed to all the chromosomes and shared 12–34% identity with each other, with a sequence length ranging from 214 to 1403 amino acids. A kinase domain in the C-terminal and a long regulatory domain in the N- terminal region were identified in most FvMAPKKKs, suggesting that the specific long regulatory domain exited in the N-terminal of the RAF subfamily may involve in specify the kinase activity and regulating protein interaction [45]. It is interesting that no FvMAPKKK was found that belonged to the ZIK subfamily, which in other species has members with a conserved GTPEFMAPE (L/V) Y signature. The absence of the characteristic ZIK subfamily feature in *F. vesca* might suggest the presence of a slightly modified RAF domain, instead of the typical ZIK domain as reported in *Vitis* [42].

As the last two steps of the MAPK cascade, MAPK and MAPKK link upstream kinases and downstream substrates. As dual-specificity kinases, MAPKKs can be activated and phosphorylated by MAPKKKs via phosphorylation of Thr/Ser residues, while MAPKKs also phosphorylate the downstream MAPKs, and activated MAPKs can phosphorylate different substrates, including other transcription factors, kinases, and cytoskeleton binding proteins [3]. The MAPK cascade protein (MAP4K-MAPKKK-MAPKK-MAPK) family is large and the functions of the various members can be complex. Though there are many members identified in the MAPKKK and MAP4K subfamilies, little is known about the MAPKs and MAPKKs. In this study, we mainly focused our gene expression pattern analysis on the *MAPK* and *MAPKK* genes. We evaluated the expression patterns of all of the predicted members of the *FvMAPK* and *FvMAPKK* subfamily in different strawberry organs and developmental stages. The expression of most of the *FvMAPK* and *FvMAPKK* genes was detected in the strawberry organs investigated, possibly reflecting their involvement in a common metabolic and/or developmental process. Among them, *FvMAPK7*, *FvMAPK10* and *FvMAPKK2* showed significantly higher expression in leaves than in other organs, and *FvMAPKK6* and *FvMAPKK7* were specifically expressed in leaves, whereas the *FvMAPK6* transcripts were not be detected in leaves, indicating different functions for divergent family members.

Stress-specific increased *MAPK* genes and kinase activity has been identified when plants are suffering from a variety of abiotic stresses, including drought, cold, high salinity, ozone, UV irradiation and oxidative stress [46,47,48,49]. Plant hormones were detected playing a role in trigger stress responses and developmental pathways with other signaling molecules [46,50,51,52]. The components of MEKK1, MKK2, MPK4, and/or MPK6 respond to drought, salt, or cold stressed were characterized in *A. thaliana* [22,47]. We studied the interaction between hormone and gene expression patterns in strawberry fruit. FvMAPK5 and FvMAPK8 belong to group II, which contains well-characterized *MAPK* genes including *AtMAPK3* and *AtMAPK6*. It has been reported that *AtMAPK3* can be activated in response to abiotic stresses and pathogens, and *AtMAPK6* also plays a role in abiotic and biotic stresses [10,39,53,54]. Similarly, we speculate that *FvMAPK5* and *FvMAPK8* are important for abiotic or biotic stress responses due to their transcriptional activation by cold and drought. FvMAPK1, FvMAPK9 and FvMAPK10 are clustered in Group I, while also includes AtMAPK4, AtMAPK5, AtMAPK11, AtMAPK12 and AtMAPK13. AtMPK4 and its upstream MAPKK, AtMKK2, are involved in both biotic and abiotic stresses. The expression patterns of *FvMAPK1*, *FvMAPK9* and *FvMAPK10* were similar in fruits but not in leaves, suggesting different functions depending on the organ [55].

In *A. thaliana*, *AtMPKK3* is up-regulated in response to ABA [46], and *FvMPKK5*, which showed the highest homology to *AtMPKK3*, showed strong activation by ABA in leaves, suggesting a similar function. Interestingly, *AtMKK1*/*AtMKK2*, which are activated by salt, drought, and cold stresses [22,23], and their ortholog *FvMAPKK3*, showed specific activation by salt stress in leaves and significant expression changes due to cold and heat stress.

## 4. Materials and Methods

### 4.1. Identification of Potential MAPK Cascade Gene Family Members in Strawberry

To identify a complete list of strawberry *MAPK* cascade genes, we used two public databases: the National Centre for Biotechnology Information (NCBI; http://www.ncbi.nlm.nih.gov/) and *F. vesca* BioView Gene Model Database (https://strawberry. plantandfood.co.nz/).

### 4.2. Phylogenetic Analysis of A. Thaliana and the Strawberry MAPK Cascade Genes

Phylogenetic analysis was performed using the MEGA 5.1 software (USA) and the neighbor-joining method. Bootstrap values were calculated for 1000 iterations [56,57]. The *A. thaliana MAPK* cascades gene family database was obtained from TAIR (The *A. thaliana* Information Resource, http://www.arabidopsis.org) and used for comparative analysis.

### 4.3. Plant Material and Fruit Pre-Treatments

*F. vesca* (“Hawaii 4”) plants were grown on a growth chamber at 22 ± 1 °C in a 13/11 h dark/light photoperiod. Fruit samples were harvested every 6 days starting of the 18 days after flowering (18, 24, 30, 36 and 42 DAF). At each developmental stage, ten representative fruits were sampled, and tissues were immediately frozen in liquid nitrogen and stored at −80 °C until further use. In order to obtain different pre-treatment experimental fruits, two stages (18 and 36 DAF) were selected for sucrose and hormone treatments. The fruits were cut in half longitudinally, and half was used as a control while the other half was used for processing. The different treatments used in this work were indole-3-acetic acid (IAA), abscisic acid (ABA) and sucrose, at a concentration of 100, 100 and 50 μM respectively. The high and low temperature was 40 °C and 4 °C. All other experiments were performed at 25 °C.

### 4.4. The Processing of Strawberry Leaves under Different Stress and Hormone Treatments 

To investigate the induced expression patterns of the *FvMAPK* and *FvMAPKK* genes in seedling leaves in response to various treatments, 30 days old soil grown seedlings were used. For salt stress treatment, seedlings were irrigated in 100 mM NaCl, collected 2, 4, 6, 8, 10 and 12 days after salt treatment of leaves, without salt stress as control. For the drought stress treatment, all plants were stopped to water after full irrigation watering, then clipping the leaves in 2, 4, 6, 8, 10 and 12 days and no drought stress as the control respectively. For the cold treatment, seedlings were maintained at 0 °C and leaf material collected at 4, 8, 12, 24, 48 and 72 h. Leaves cultivated in a growth chamber at 22 ± 1 °C were used as controls. For the hormone treatments, plants were sprayed with solutions containing 100 μM ABA or 100 μM IAA, then leaves were collected after 2, 4, 6, 8, 10 and 12 days. Plants that had not been sprayed served as the controls. Samples were immediately frozen in liquid nitrogen, and stored at −80 °C.

### 4.5. RNA Extraction and Real-Time PCR Analysis

Total RNA was isolated from strawberry fruits harvested at 18, 24, 30, 36 and 42 DAF using the Plant RNA Kit (Omega, USA) according to the manufacturer’s instructions, and total RNA was reverse transcribed into cDNA using the Invitrogen reverse transcription kit (SuperScriptШ Reverse Transcriptase, USA). Real-time PCR was performed to confirm gene expression patterns using a Light Cycler^®^ 96 SW1.1 Real Time PCR System (Roche, Germany), with SYBR-Green (Takara, Dalian, China). The primer sequences used are designed based on gene sequences and the Beacon designer software and are shown in Table 2 in this study. Each reaction consists of 5 μL SYBR, 3.5 μL ddH_2_O, 1 μL diluted template (1 μL of the generated first-strand cDNA diluted by 9 μL ddH_2_O) and 0.25 μL of each of two gene specific primers. Thermal cycling conditions were 95 °C for 10 min, then 40 cycles at 95 °C for 20 s, 54 °C for 20 s, 72 °C for 20 s.

## 5. Conclusions

MAPK cascades are responsible for protein phosphorylation during signal transduction events, and typically classify as three protein kinases: MAPK, MAPK kinase, and MAPK kinase kinase. Our study provides a comprehensive overview of the 12 *FvMAPK*, 7 *FvMAPKK*, 73 *FvMAPKKK* and 1 *FvMAPKKKK* genes identified in the *F. vesca* genome. The identification of MAPK cascade proteins and a comparative analysis with the *A. thaliana* MAPK cascade proteins indicated that the MAPK cascade genes have been conserved during evolution and suggests that MAPK cascades play vital roles in fruit development and in responses to abiotic stresses and hormone signal transduction. Among these genes, *FvMAPK7*, *FvMAPK10* and *FvMAPKK2* showed significantly higher expression in leaves than in other organs, and *FvMAPK11* and *FvMAPKK2* have an up-regulation and significantly improved in the later stage of fruit development. For various hormones and stresses, cold stress has great influence on gene expression. This information will be helpful in the physiological and biochemical functional characterization of the MAPK cascades in strawberry.

## Figures and Tables

**Table 1:** MAPK cascade proteins identified in the *Fragaria vesca* genome.

Gene Name	Gene ID	Chr	Location	ORF (aa)	mW (kDa)	pI	Instability Index	Ai	GRAVY	Number of Exons
*MAPKs*										
*MAPK1*	101290762	5	1784699..1788845	377	43.24	6.4	46.14	90.53	−0.37	6
*MAPK2*	101294541	3	11156420..11160717	609	69.37	9.26	32.32	83.92	−0.44	10
*MAPK3*	101295052	5	10188960..10193438	691	78.45	7.86	44.65	75.96	−0.64	11
*MAPK4*	101295411	4	13067191..13073043	561	63.82	8.73	36.24	77.4	−0.48	10
*MAPK5*	101297368	1	1081422..1084925	391	44.71	5.65	39.58	89.82	−0.28	5
*MAPK6*	101300335	4	18603202..18607991	580	66.33	7.07	38.18	80.53	−0.55	12
*MAPK7*	101306152	6	26819611..26825467	618	70.68	9.23	37.11	81.12	−0.47	10
*MAPK8*	101306313	3	19690549..19693371	371	42.71	5.62	39.46	93.32	−0.26	6
*MAPK9*	101308652	3	19870360..19873988	365	41.85	5.05	42.04	95.34	−0.28	6
*MAPK10*	101313449	7	4574723..4578939	373	42.67	6.08	43.9	89.38	−0.33	6
*MAPK11*	101313547	1	9041132..9043978	370	42.64	8.32	33.43	94.32	−0.24	3
*MAPK12*	101314433	2	32254545..32257169	372	42.64	6.09	38.46	95.13	−0.25	3
*MAPKKs*										
*MAPKK1*	101296494	5	21018101..21019144	347	38.73	5.64	58.27	86.54	−0.19	1
*MAPKK2*	101298915	6	26457958..26460120	365	40.37	9.28	60.36	76.68	−0.44	1
*MAPKK3*	101299988	1	5116387..5119659	355	39.86	6.14	51.1	95.75	−0.18	8
*MAPKK4*	101300536	5	9858540..9860210	325	36.28	8.01	58.45	82.52	−0.26	1
*MAPKK5*	101302771	6	37368876..37372756	518	57.53	5.34	45.36	89.63	−0.15	9
*MAPKK6*	101309937	5	1450708..1451997	244	26.72	6.44	56.61	80.29	−0.31	1
*MAPKK7*	101310227	5	1459329..1461416	350	38.86	6.01	67.41	76.06	−0.35	1
*MAPKKKs*										
*MAPKKK1*	101291312	2	32932997..32939667	714	78.16	9.58	56.43	66.79	−0.55	10
*MAPKKK2*	101291614	3	13958286..13959565	311	34.68	5.59	39.15	79.9	−0.31	1
*MAPKKK3*	101291671	1	4101379..4109515	973	108.54	7.03	45.49	81.31	−0.42	15
*MAPKKK4*	101291740	6	36971098..36974966	384	42.80	6.76	36.76	75.62	−0.49	6
*MAPKKK5*	101292378	2	18357934..18361624	278	31.77	9.66	43.47	96.12	−0.04	8
*MAPKKK6*	101292778	3	8405027..8408729	539	61.14	9.57	50.83	76.36	−0.57	4
*MAPKKK7*	101292844	1	4173320..4175043	265	29.70	5.77	46.62	83.47	−0.40	1
*MAPKKK8*	101293476	4	7105385..7111294	390	43.69	6.48	32.42	79.23	−0.44	7
*MAPKKK9*	101294141	2	2588978..2594536	1126	125.14	5.80	51.66	74.17	−0.58	9
*MAPKKK10*	101294311	7	2095625..2102937	1221	135.58	5.47	47.97	72.09	−0.61	9
*MAPKKK11*	101294663	5	16790800..16799279	511	55.61	8.61	52.95	79.77	−0.45	8
*MAPKKK12*	101294665	5	24653764..24659548	591	65.24	5.68	54.8	70.93	−0.59	9
*MAPKKK13*	101294687	6	25086652..25087498	214	24.04	6.34	49.53	82.9	−0.47	2
*MAPKKK14*	101295466	7	2167754..2172789	690	76.10	6.23	37.12	84.51	−0.24	15
*MAPKKK15*	101295905	3	26006132..26009750	415	46.98	7.91	40.22	86.7	−0.43	12
*MAPKKK16*	101296046	7	2179758..2185318	678	75.20	5.52	39.59	87.45	−0.22	15
*MAPKKK17*	101296626	2	17778744..17784528	570	63.92	5.88	55.83	84.63	−0.49	16
*MAPKKK18*	101297008	1	11236088..11240295	625	68.99	9.27	67.35	68.82	−0.54	11
*MAPKKK19*	101297162	6	5243004..5248949	670	74.51	5.97	57.02	71.43	−0.53	17
*MAPKKK20*	101298508	4	17684149..17687733	434	49.07	7.79	36.92	84.06	−0.48	11
*MAPKKK21*	101298797	5	2958127..2962070	404	44.58	7.51	37.14	80.37	−0.39	6
*MAPKKK22*	101298822	6	35785690..35790732	457	51.78	6.10	44.73	84.07	−0.46	13
*MAPKKK23*	101299949	4	8553004..8558770	1092	121.56	5.55	43.15	72.31	−0.59	9
*MAPKKK24*	101299957	5	2999007..3002862	347	39.54	8.04	52.15	82.65	−0.33	3
*MAPKKK25*	101300060	5	1036941..1041266	697	78.06	6.49	44.46	77.91	−0.58	14
*MAPKKK26*	101300175	1	1133811..1138366	475	53.79	9.10	44.71	86.82	−0.45	13
*MAPKKK27*	101300188	7	404217..406592	344	37.94	5.76	36.34	88.46	−0.07	1
*MAPKKK28*	101300232	3	626166..628364	407	44.97	4.89	47.56	75.92	−0.30	1
*MAPKKK29*	101300748	6	105416..107101	426	47.79	8.88	45.4	80.61	−0.45	1
*MAPKKK30*	101302206	1	3358613..3363681	995	109.61	8.87	43.56	77.84	−0.47	8
*MAPKKK31*	101302247	3	26940964..26947028	717	79.93	5.70	43.53	77.98	−0.33	10
*MAPKKK32*	101302307	1	10029166..10031248	415	45.28	5.03	46.91	84.36	−0.14	1
*MAPKKK33*	101302624	3	4232964..4238052	458	51.96	7.59	49.66	90.72	−0.41	12
*MAPKKK34*	101302797	7	19633448..19634707	325	36.69	8.72	39.02	80.98	−0.36	1
*MAPKKK35*	101303378	7	19639527..19640520	322	36.61	8.80	40.45	87.17	−0.42	1
*MAPKKK36*	101303395	2	22992935..22998826	555	61.75	6.30	47.28	86.74	−0.36	16
*MAPKKK37*	101303762	2	14719795..14732427	554	62.13	5.37	42.28	87.27	−0.39	16
*MAPKKK38*	101303943	7	620946..625977	343	37.74	6.05	38.37	87.03	−0.10	3
*MAPKKK39*	101304212	6	25909877..25910863	328	36.85	8.61	42.91	89.45	−0.25	1
*MAPKKK40*	101304439	2	16018344..16021537	400	44.29	7.53	30.1	75.07	−0.42	6
*MAPKKK41*	101305170	6	21759476..21761269	309	34.91	4.67	38.89	94.4	−0.13	4
*MAPKKK42*	101305413	7	5759511..5762791	444	49.43	6.92	55.58	82.79	−0.26	10
*MAPKKK43*	101305446	3	25233157..25239380	777	86.22	6.83	50.65	68.51	−0.66	13
*MAPKKK44*	101305461	4	12404607..12409591	677	74.37	7.24	47.43	74.93	−0.44	17
*MAPKKK45*	101305547	3	15016023..15017869	447	49.64	4.50	50.48	80.02	−0.19	1
*MAPKKK46*	101305739	3	5762142..5770103	1323	145.89	5.11	46.54	75.92	−0.56	9
*MAPKKK47*	101305774	6	13132618..13140647	845	93.61	5.77	44.72	81.34	−0.37	16
*MAPKKK48*	101305797	1	2243252..2248966	1403	152.80	5.25	47.01	73.02	−0.52	10
*MAPKKK49*	101307123	5	17495323..17497199	405	45.08	5.36	55.35	76.07	−0.16	2
*MAPKKK50*	101307418	6	37601874..37604741	346	38.84	7.21	42.97	84.91	−0.24	6
*MAPKKK51*	101307975	3	13141362..13142841	488	53.77	4.85	49.32	79.04	−0.15	1
*MAPKKK52*	101308436	2	20472173..20477216	927	101.52	5.36	43.52	83.95	−0.32	13
*MAPKKK53*	101308592	6	15883396..15899615	765	85.91	5.77	51.23	80.58	−0.41	18
*MAPKKK54*	101308868	5	1573420..1580407	902	98.41	9.44	60.15	64.01	−0.60	12
*MAPKKK55*	101309867	1	8084450..8090390	572	64.91	5.69	49.85	83.5	−0.43	16
*MAPKKK56*	101309911	3	949163..951911	374	42.66	8.96	46.65	81.1	−0.42	3
*MAPKKK57*	101310026	5	10174217..10182364	1034	112.94	5.53	48.91	81.21	−0.44	13
*MAPKKK58*	101310764	7	22365118..22371514	903	97.23	9.71	65.72	62.98	−0.59	12
*MAPKKK59*	101312454	5	26796369..26801049	390	44.24	5.57	43.6	83.49	−0.40	6
*MAPKKK60*	101312659	6	20974416..20976740	433	49.51	5.10	37.22	83.79	−0.48	5
*MAPKKK61*	101312816	3	25255805..25265535	794	89.06	6.09	50.14	76.49	−0.45	17
*MAPKKK62*	101312898	2	9859412..9866665	1192	130.83	5.15	48.2	80.05	−0.44	9
*MAPKKK63*	101313105	3	25269938..25276589	710	80.27	7.32	41.81	78.39	−0.48	13
*MAPKKK64*	101313212	5	5764644..5768035	350	39.78	6.43	46.69	83.09	−0.30	7
*MAPKKK65*	101313251	1	16064274..16069495	618	67.06	9.22	52.62	67.38	−0.53	11
*MAPKKK66*	101313299	3	26914066..26919397	239	27.17	9.23	30.57	96.69	−0.21	11
*MAPKKK67*	101315161	6	6707178..6711235	358	40.09	9.03	39.93	88.02	−0.30	6
*MAPKKK68*	101315443	6	93407..100195	918	100.48	5.91	44.43	77.24	−0.45	13
*MAPKKK69*	105349270	7	5764174..5768902	438	47.17	10.09	59.37	77.92	−0.48	10
*MAPKKK70*	105349796	2	18355556..18356787	219	24.73	8.73	42.83	89.45	−0.21	4
*MAPKKK71*	105351460	1	10823118..10824804	351	39.06	4.97	47.76	73.59	−0.36	1
*MAPKKK72*	105351574	5	13162396..13164156	298	33.63	6.84	50.69	82.08	−0.43	1
*MAPKKK73*	105353223	7	5144013..5149034	682	77.82	8.15	44.6	87.76	−0.40	13
*MAPKKKKs*										
*MAP4K1*	101308962	4	17222313..17230075	821	90.20	5.21	47.82	71.06	−0.54	18

**Table 2:** Primer sequence information.

Gene	Sequences 5′→3′	Annealing Temperature
*MAPK1*	F: TAGCAAGAACAACATCCGAGAC	54 °C
	R: GCTCCAGGCGACATATTAGG	
*MAPK2*	F: ACACCTACACTAGAGACCATC	54 °C
	R: TGCCTTCCGTTCCATTCAT	
*MAPK3*	F: CCTTGGTGGACGGTGTTC	54 °C
	R: GGTGGTGGTGGATTGTGG	
*MAPK4*	F: CTTATCTTGAGGAGCACTATGGAA	54 °C
	R: CGTAATACTGAGCCGACAACT	
*MAPK5*	F: GCGGCAGATTCATCCAGTA	54 °C
	R: TGACAATGCTCCTCAGATAGA	
*MAPK6*	F: GCATCTCCACTTCTACATCTTCA	54 °C
	R: TGCCAATCACTTCTTGTATCTCA	
*MAPK7*	F: ACCGAAGGACGCTTGTTAG	54 °C
	R: AGCAGCAGCAGAACCAAT	
*MAPK8*	F: CATCGTCTGCTCGGTGTT	54 °C
	R: GCTCGGCTTCAAGTCTCTAT	
*MAPK9*	F: CCACCAGATAAGGAGAACTTCAAT	54 °C
	R: CGATACCAACGAGTGACAACA	
*MAPK10*	F: AGTGATAATGCCCGAAGATATGT	54 °C
	R: GGATTGAACTTGACCGACTC	
*MAPK11*	F: GAGAGGAGCATACGGTGTTG	54 °C
	R: AGTTAGCATTGATGAGCAGGTT	
*MAPK12*	F: CTGTGCTGCGATAACTATGGA	54 °C
	R: GGTGTTGGAGTGCTTCAG	
*MAPKK1*	F: TGACTCTCCAGGCATTATTGAA	54 °C
	R: GCCACGCCAGAGATTACC	
*MAPKK2*	F: CTCCACAGACGGCACATC	54 °C
	R: GGCGGCTGAGACATACAAAT	
*MAPKK3*	F: GAAAGGAAGTGGTGGTGTAGT	54 °C
	R: GCAAGATATGGTTCAAGAATTGTCT	
*MAPKK4*	F: ACTGCTCCTCCTCGTCTT	54 °C
	R: CGTGCCTTGCTGTTTGA	
*MAPKK5*	F: GGATGAAGGACTTGGCAGAT	54 °C
	R: AACACCGTCTCCACATATAAGG	
*MAPKK6*	F: CCACCTCCACCACTTACG	54 °C
	R: GGCGATCTTGACCTCATTCT	
*MAPKK7*	F: CTCCACCACTTACGCTCTC	54 °C
	R: GACCTCGTTCTTGTTGTTCATT	
*Actin*	F: TGGGTTTGCTGGAGATGAT	54 °C
	R: CAGTTAGGAGAACTGGGTGC	
